# Supplementary material for: Transcription factor prediction using protein 3D secondary structures
Source: Bioinformatics. 2025 Jan 9;41(1):btae762. doi: 10.1093/bioinformatics/btae762 (PMC11769678; doi:10.1093/bioinformatics/btae762)
Supplement: btae762_Supplementary_Data [file btae762_supplementary_data.zip › 2963f_Supplement.pdf]

Supplementary materials: Transcription factor prediction using protein 3D secondary structures

Jeanine Liebold,[1,2†] **Fabian Neuhaus**,[1,†] **Janina Geiser**,[1] **Stefan Kurtz**,[2] **Jan Baumbach**[1,3] **and Khalique Newaz**[1,4,*]

[1]Institute for Computational Systems Biology, Universität Hamburg, Albert-Einstein-Ring 8-10, 22761 Hamburg, Germany, [2]Faculty of Mathematics, Informatics and Natural Sciences, ZBH - Center for Bioinformatics, Universität Hamburg, Albert-Einstein-Ring 8-10, 22761 Hamburg, Germany, [3]Department of Mathematics and Computer Science, University of Southern Denmark, Campusvej 55, 5230 Odense, Denmark and [4]Center for Data and Computing in Natural Sciences, Universität Hamburg, Albert-Einstein-Ring 8-10, 22761 Hamburg, Germany

[*]Corresponding author, [†]Equal contribution

I Supplementary sections

S1. Information regarding 3D secondary structural data from PDB

To evaluate the possibility of using experimentally determined structures from the Protein Data Bank (PDB) (Berman et al., 2000), we analyze the PDB data as follows. We consider all UniProt proteins with PDB entries having 3D resolutions of $\leq 3\mathring{A}$, resulting in 25 442 proteins. Because one protein can have multiple PDB chain entries, for each of the 25 442 proteins, we select the PDB chain entry with the highest number of 3D resolved residues (i.e., the PDB chain with the maximum number of residues with secondary structural information). Then, for each protein, to quantify the fraction of residues with secondary structural information (named "3D residue coverage"), we divide the number of 3D structurally resolved residues of a protein with its total number of residues.

We find that most proteins have ≤ 0.5 3D residue coverage (Supplementary Figure S1). Because the focus of this study is to build a TF prediction model, we need sufficient number of TFs to train such a model. We find that, among the 25 442 proteins, there are 1 147 TFs. However, only 295 out of the 1 147 (i.e., $\sim$26%) TFs have a 3D residue coverage of ≥ 0.5, and this percentage decreases drastically with an increase in the 3D residue coverage value (Supplementary Table S1). This analysis shows the limited availability of the PDB data to build a 3D structure-based machine learning model for TF prediction.

S2. Datasets based on allAF proteins

Similar to reliableAF (Section 2.5 in the main paper), we create six datasets, i.e., $D(a, z, s)$ where $z \in \{nr, r\}$ and $s \in \{3, 5, 10\}$, for allAF. Out of all 524 674 allAF proteins, there are 19 014 TFs and 505 660 non-TFs. To remove the class imbalance problem, we take three random samples from all 505 660 non-TFs, such that the number of non-TFs is either 3, 5, or 10 times more than the number of TFs, resulting in three datasets $D(a, r, 3)$, $D(a, r, 5)$, and $D(a, r, 10)$. Additionally, we remove sequence redundancy among the 524 674 proteins in the similar manner as we do for reliableAF, which results in 61 025 (with 3 798 TFs and 57 227 non-TFs) proteins. Then, we remove the class imbalance problem as above from the dataset with sequence non-redundant proteins, resulting in three datasets named $D(a, nr, 3)$, $D(a, nr, 5)$, and $D(a, nr, 10)$, see Figure 2 in the main paper.

S3. Performance of the TF prediction methods on datasets with only human proteins

All of our results in the main paper are based on proteins from all available species combined. To evaluate whether the results in the main paper also hold for individual species, we do the following preliminary analysis. We use all 16 972 human proteins from allAF. This set consists of 1 487 TFs and 15 485 non-TFs. Then, similar to how we create three datasets from allAF with potential sequence redundancy (Supplementary Section S2), we create three datasets with only human proteins from allAF, i.e., $D(ha, r, s)$ where $s \in \{3, 5, 10\}$ and 'ha' denotes that the proteins are from humans. We find that StrucTFactor performs similarly to DeepTFactor but better than DeepReg (Supplementary Figure S4). Qualitatively, these results are comparable to the results of the main paper that are based on potential sequence redundant datasets from allAF proteins comprising all species combined, i.e., for datasets $D(a, r, 3)$, $D(a, r, 5)$, and $D(a, r, 10)$ in Supplementary Table S5. Note that although the three human datasets (i.e., $D(ha, r, s)$ with $s \in \{3, 5, 10\}$) inherently removes orthologs, they could still have paralogs that share high sequence and structural similarity across training and testing sets, and thus could bias the performance evaluation results. To remove such potential paralogs from these datasets, similar to our study based on all species combined, first, given the 16 972 human proteins in allAF, we only retain those human proteins that have pairwise sequence identities of less than 30%. This results in 10 868 sequence non-redundant human proteins (754 TFs and 10 114 non-TFs). Then, similar to how we create three sequence non-redundant datasets from allAF proteins (Supplementary Section S2), we create three datasets with only sequence non-redundant human proteins from allAF, i.e., $D(ha, nr, s)$ where $s \in \{3, 5, 10\}$, and 'ha' denotes that the proteins are from human. On these datasets, we find that StrucTFactor outperforms both DeepTFactor and DeepReg by a relatively larger margins (Supplementary Figure S5). These results are qualitatively comparable to the results for datasets $D(a, nr, 3)$, $D(a, nr, 5)$, and $D(a, nr, 10)$ in Supplementary Table S5.

II Supplementary figures

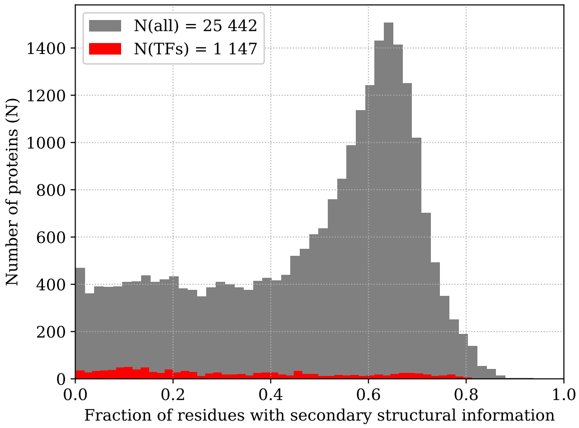

Figure S1: Distribution of fractions of protein residues with secondary structural information (i.e., 3D residue coverage) from PDB. The grey bars represent the distribution of 3D residue coverage for all 25 442 UniProt proteins with a PDB entry having the 3D resolution of $\leq 3\mathring{A}$. The red bars show the subset of 25 442 (i.e., 1 147) proteins that are TFs.

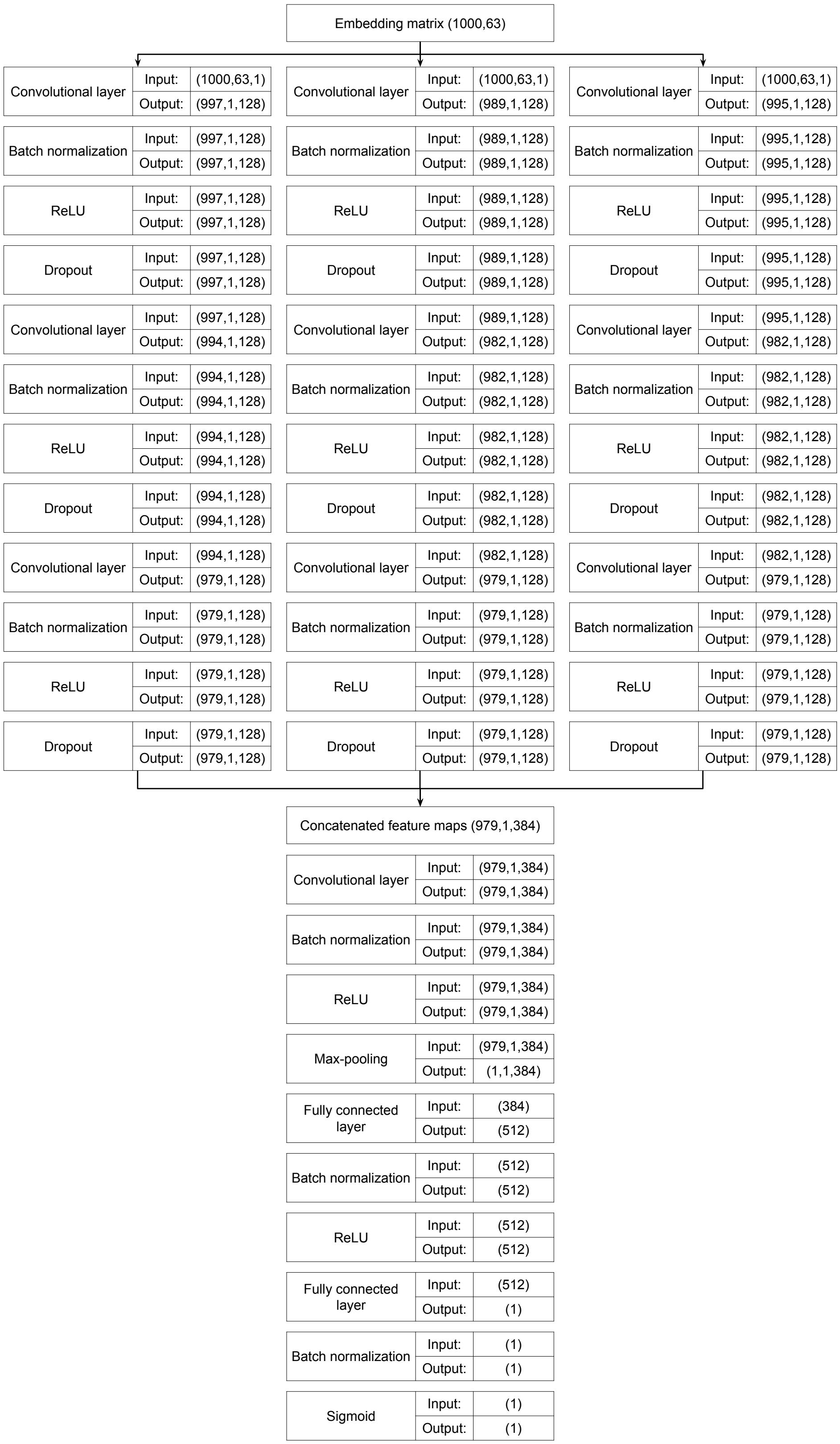

Figure S2: Deep learning architecture of StrucTFactor. Figure adapted from (Kim et al., 2021).

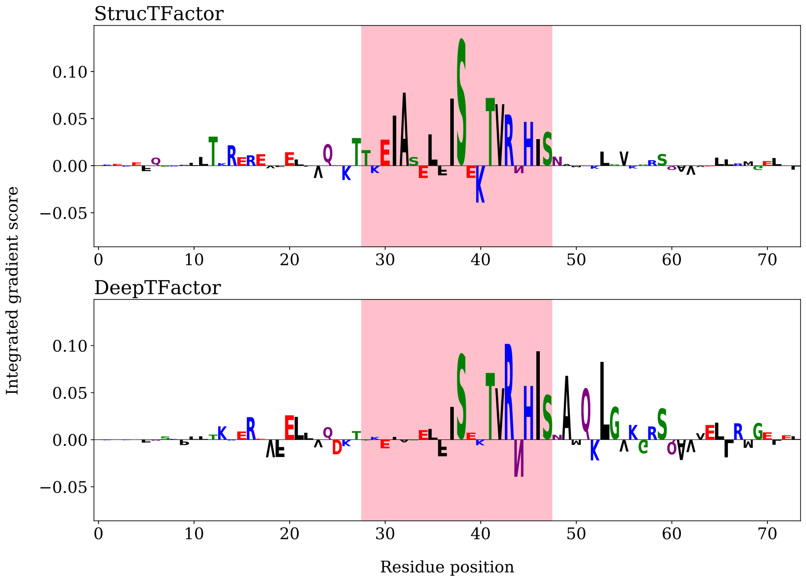

Figure S3: Integrated gradient score of each sequence position of the Spore germination protein GerE (UniProt ID *P11470*). This protein achieves the highest TF prediction score by both StrucTFactor and DeepTFactor. We highlight the known DBD region in pink (ground truth knowledge and not part of the predictions). For each position along the protein sequence on the X-axes, the corresponding integrated gradient score is represented on the Y-axes, quantified by the size and orientation of the amino acid letter.

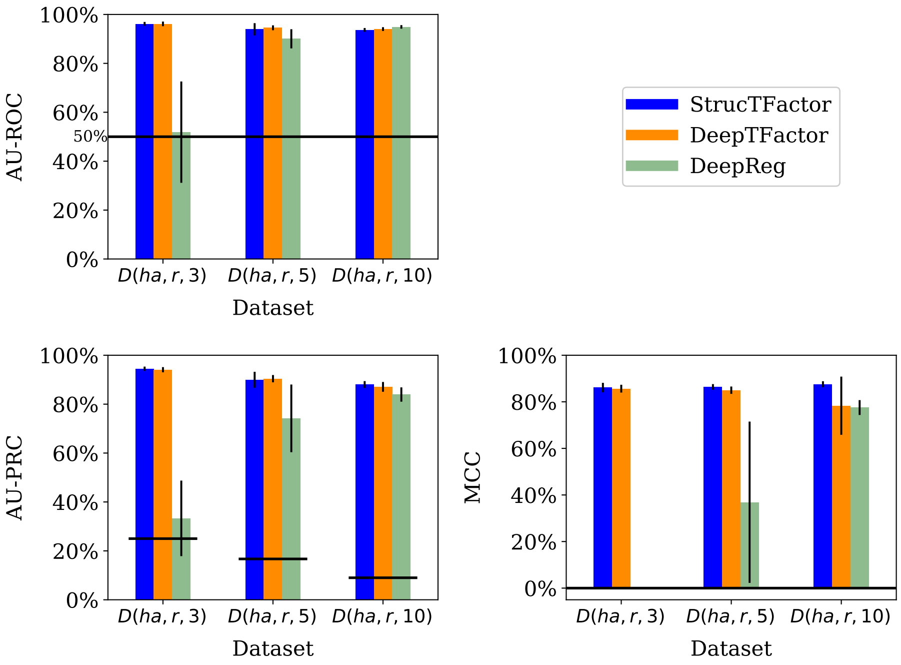

Figure S4: Performances of StrucTFactor (blue), DeepTFactor (orange), and DeepReg (green) based on AU-ROC (upper left panel), AU-PRC (lower left panel) and MCC (lower right panel) for three datasets created using human proteins from allAF. In the figure, the three datasets are represented as $D(ha, r, s)$ where $s \in \{3, 5, 10\}$ denotes the ratio of non-TFs vs. TFs, 'ha' stands for human proteins from allAF, 'r' indicates that the sequences in a dataset could be sequence redundant. The number of TFs (1 487) in each dataset is the same. Height of a bar represents the average performance over the five independent test sets, while the vertical line on a bar represents the corresponding standard deviation. The horizontal lines for AU-ROC and AU-PRC indicate the expected performance for a dataset using a random classifier.

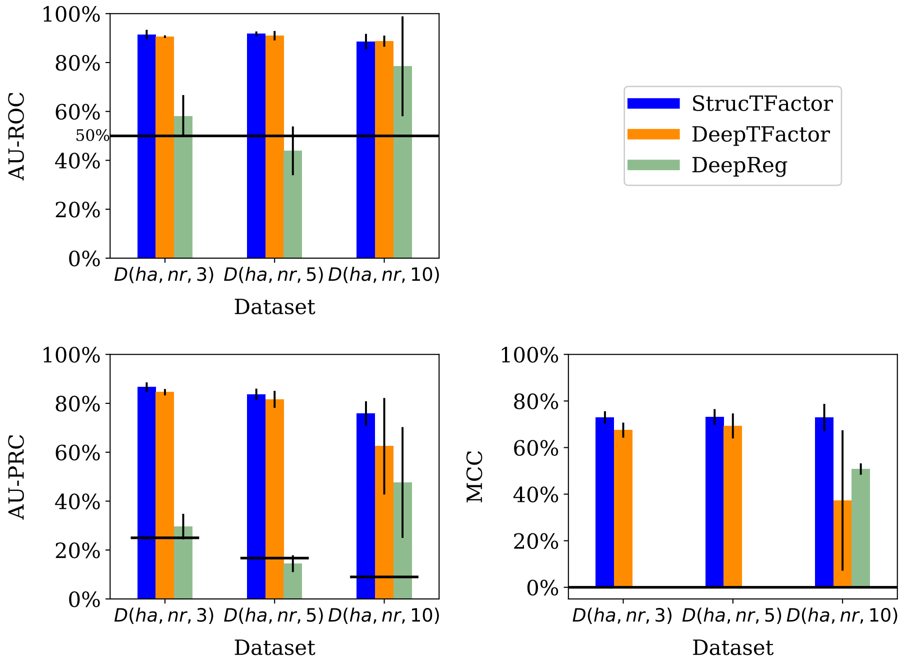

Figure S5: Performances of StrucTFactor (blue), DeepTFactor (orange), and DeepReg (green) based on AU-ROC (upper left panel), AU-PRC (lower left panel) and MCC (lower right panel) for three datasets created using human proteins from allAF. In the figure, the three datasets are represented as $D(ha, nr, s)$ where $s \in \{3, 5, 10\}$ denotes the ratio of non-TFs vs. TFs, 'ha' stands for human proteins from allAF, 'nr' indicates that the sequences are potentially sequence non-redundant. The number of TFs (754) in each dataset is the same. Height of a bar represents the average performance over the five independent test sets, while the vertical line on a bar represents the corresponding standard deviation. The horizontal lines for AU-ROC and AU-PRC indicate the expected performance for a dataset using a random classifier.

III Supplementary tables

Table S1 Fraction of residues in a protein with secondary structural information. We show results for all considered PDB proteins and all considered PDB TFs.

Fraction of residues in a protein with secondary structural information ≥ x	x = 0.0	x = 0.5	x = 0.6	x = 0.7	x = 0.8	x = 0.9
Number of all considered UniProt proteins with sufficient 3D structural resolution	25 442	14 439	9 747	2 664	295	10
Number of all considered UniProt TFs with sufficient 3D structural resolution	1 147	295	217	91	5	0

Table S2 Summary of the GO terms used for labeling proteins as TFs versus non-TFs, adopted from (Kim et al., 2021) and (Ledesma-Dominguez et al., 2024).

Type	GO Terms	Description
Transcription factor	GO:0000976	Transcription regulatory region sequence-specific DNA binding
Transcription factor	GO:0000977	RNA polymerase II transcription regulatory region sequence-specific DNA binding
Transcription factor	GO:0000978	RNA polymerase II cis-regulatory region sequence-specific DNA binding
Transcription factor	GO:0000979	RNA polymerase II core promoter sequence-specific DNA binding
Transcription factor	GO:0000981	DNA-binding Transcription factor activity, RNA polymerase II-specific
Transcription factor	GO:0000984	Bacterial-type RNA polymerase transcription regulatory region sequence-specific DNA binding
Transcription factor	GO:0000985	Bacterial-type RNA polymerase core promoter sequence-specific DNA binding
Transcription factor	GO:0000986	Bacterial-type cis-regulatory region sequence-specific DNA binding
Transcription factor	GO:0000987	cis-regulatory region sequence-specific DNA binding
Transcription factor	GO:0000992	RNA polymerase III cis-regulatory region sequence-specific DNA binding
Transcription factor	GO:0000995	RNA polymerase III general transcription initiation factor activity
Transcription factor	GO:0001046	Core promoter sequence-specific DNA binding
Transcription factor	GO:0001163	RNA polymerase I transcription regulatory region sequence-specific DNA binding
Transcription factor	GO:0001164	RNA polymerase I core promoter sequence-specific DNA binding
Transcription factor	GO:0001165	RNA polymerase I cis-regulatory region sequence-specific DNA binding
Transcription factor	GO:0001216	DNA-binding transcription activator activity
Transcription factor	GO:0001227	DNA-binding transcription repressor activity, RNA polymerase II-specific
Transcription factor	GO:0003700	DNA-binding TF activity
Transcription factor	GO:0034246	Mitochondrial sequence-specific DNA-binding TF activity
Transcription factor	GO:0098531	Ligand-activated TF activity
Transcription factor	GO:0106250	DNA-binding transcription repressor activity, RNA polymerase III-specific
Transcription regulation	GO:0001228	DNA-binding transcription activator activity, RNA polymerase II-specific
Transcription regulation	GO:0006351	Transcription, DNA-templated
Transcription regulation	GO:0006355	Regulation of transcription, DNA-templated
Transcription regulation	GO:0043433	Negative regulation of DNA-binding TF activity
Transcription regulation	GO:0045892	Negative regulation of transcription, DNA-templated
Transcription regulation	GO:0045893	Positive regulation of transcription, DNA-templated
Transcription regulation	GO:0051090	Regulation of DNA-binding TF activity
Transcription regulation	GO:0051091	Positive regulation of DNA-binding TF activity
Transcription regulation	GO:2000142	Regulation of DNA-templated transcription, initiation
Transcription regulation	GO:2000143	Negative regulation of DNA-templated transcription, initiation
Transcription regulation	GO:2000144	Positive regulation of DNA-templated transcription, initiation
DNA binding	GO:0003677	DNA binding
DNA binding	GO:0008301	DNA binding, bending
DNA binding	GO:0043565	Sequence-specific DNA binding
DNA binding	GO:0050692	DNA binding domain binding

Table S3 Performances of StrucTFactor, DeepTFactor, and DeepReg on the dataset $D(rl, nr, 3)$. For each method, the results are shown for each of the five test sets individually as well as for the average of the five test sets (last column named *Mean*). The best performing method for a given test set or for the average of the five test sets is in bold font. DeepReg predicts all proteins as non-TFs, which results in undefined ("nan") MCC scores.

	AU-PRC					
CV-Fold	1	2	3	4	5	Mean
StrucTFactor	**0.8388**	**0.8556**	**0.7839**	**0.8613**	**0.8564**	**0.8392**
DeepTFactor	0.7567	0.7256	0.7489	0.7166	0.7301	0.7356
DeepReg	0.2057	0.3602	0.3951	0.2144	0.2666	0.2884
	MCC					
CV-Fold	1	2	3	4	5	Mean
StrucTFactor	**0.7270**	**0.6739**	**0.5866**	**0.7440**	**0.6975**	**0.6858**
DeepTFactor	0.6122	0.5630	0.5395	0.6360	0.5308	0.5763
DeepReg	nan	nan	nan	nan	nan	nan
	AU-ROC					
CV-Fold	1	2	3	4	5	Mean
StrucTFactor	**0.9101**	**0.9198**	**0.8678**	**0.9176**	**0.9102**	**0.9051**
DeepTFactor	0.8971	0.8917	0.8621	0.8768	0.8530	0.8761
DeepReg	0.4209	0.6346	0.7673	0.4502	0.5550	0.5656

Table S4 Performances of StrucTFactor, DeepTFactor, and DeepReg on all data from the reliable non-redundant set of proteins containing 20,249 non-TFs and 635 TFs. Given a dataset, the results are shown as the average of the five test sets. The best average performance value corresponding to each metric is in bold font.

	AU-PRC	MCC	AU-ROC
StrucTFactor	**0.5490**	**0.5654**	**0.8262**
DeepTFactor	0.4641	0.5207	0.7536
DeepReg	0.1000	0.1658	0.6077

Table S5 Variation in the performances of StrucTFactor, DeepTFactor, and DeepReg on different sequence redundancies and class assignment ratios for our 12 datasets $D(x, z, s)$ where $x \in \{rl, a\}$, $z \in \{nr, r\}$, and $s \in \{3, 5, 10\}$ (see Section 2.5 and Figure 2 in the main paper for details). Given a dataset, the results are shown as average of the five test sets. Given a dataset and a performance measure, the best method performance is in bold font.

$D(x, z, s)$	$x = rl$ $z = nr$ $s = 3$	$x = rl$ $z = nr$ $s = 5$	$x = rl$ $z = nr$ $s = 10$	$x = rl$ $z = r$ $s = 3$	$x = rl$ $z = r$ $s = 5$	$x = rl$ $z = r$ $s = 10$	$x = a$ $z = nr$ $s = 3$	$x = a$ $z = nr$ $s = 5$	$x = a$ $z = nr$ $s = 10$	$x = a$ $z = r$ $s = 3$	$x = a$ $z = r$ $s = 5$	$x = a$ $z = r$ $s = 10$
	AU-PRC											
StrucTFactor	**0.8392**	**0.8158**	**0.7322**	**0.9876**	**0.9890**	**0.9844**	**0.8601**	**0.8225**	**0.7892**	**0.9815**	**0.9766**	0.9691
DeepTFactor	0.7356	0.7332	0.6815	0.9858	0.9869	0.9811	0.8354	0.8118	0.7449	0.9784	0.9745	**0.9702**
DeepReg	0.2884	0.1806	0.2715	0.9755	0.9426	0.9708	0.7764	0.7125	0.6573	0.9684	0.9622	0.9505
	MCC											
StrucTFactor	**0.6858**	**0.7190**	**0.6923**	**0.9747**	**0.9737**	**0.9718**	**0.7391**	**0.7394**	**0.7527**	**0.9514**	**0.9477**	**0.9484**
DeepTFactor	0.5763	0.5922	0.5741	0.9653	0.9694	0.9658	0.6850	0.7102	0.7075	0.9381	0.9439	0.9434
DeepReg	nan	0.0264	0.2574	0.8775	0.8507	0.9108	0.5417	0.5561	0.5950	0.8905	0.8826	0.9066
	AU-ROC											
StrucTFactor	**0.9051**	**0.9030**	**0.8988**	**0.9925**	**0.9941**	**0.9920**	**0.9114**	**0.9121**	**0.9069**	**0.9883**	**0.9880**	0.9829
DeepTFactor	0.8761	0.8761	0.8859	0.9914	0.9921	0.9908	0.9043	0.9028	0.8852	0.9870	0.9866	**0.9865**
DeepReg	0.5656	0.5516	0.7559	0.9877	0.9820	0.9900	0.8873	0.8838	0.8969	0.9835	0.9856	0.9850

References

H. M. Berman et al. The Protein Data Bank. *Nucleic Acids Research*, 28(1):235–242, 01 2000.

G. B. Kim et al. DeepTFactor: A deep learning-based tool for the prediction of transcription factors. *Proceedings of the National Academy of Sciences*, 118(2):e2021171118, 2021.

L. Ledesma-Dominguez et al. DeepReg: a deep learning hybrid model for predicting transcription factors in eukaryotic and prokaryotic genomes. *Scientific Reports*, 14(1), Apr. 2024.
